# Supplementary material for: Microbiome Composition and Microbial Community Structure in Mosquito Vectors Aedes aegypti and Aedes albopictus in Northeastern Thailand, a Dengue-Endemic Area
Source: Insects. 2023 Feb 13;14(2):184. doi: 10.3390/insects14020184 (PMC9961164; doi:10.3390/insects14020184)
Supplement: Supplementary file 1 [file insects-14-00184-s001.zip › insects-2193863 Figure S1.pdf]

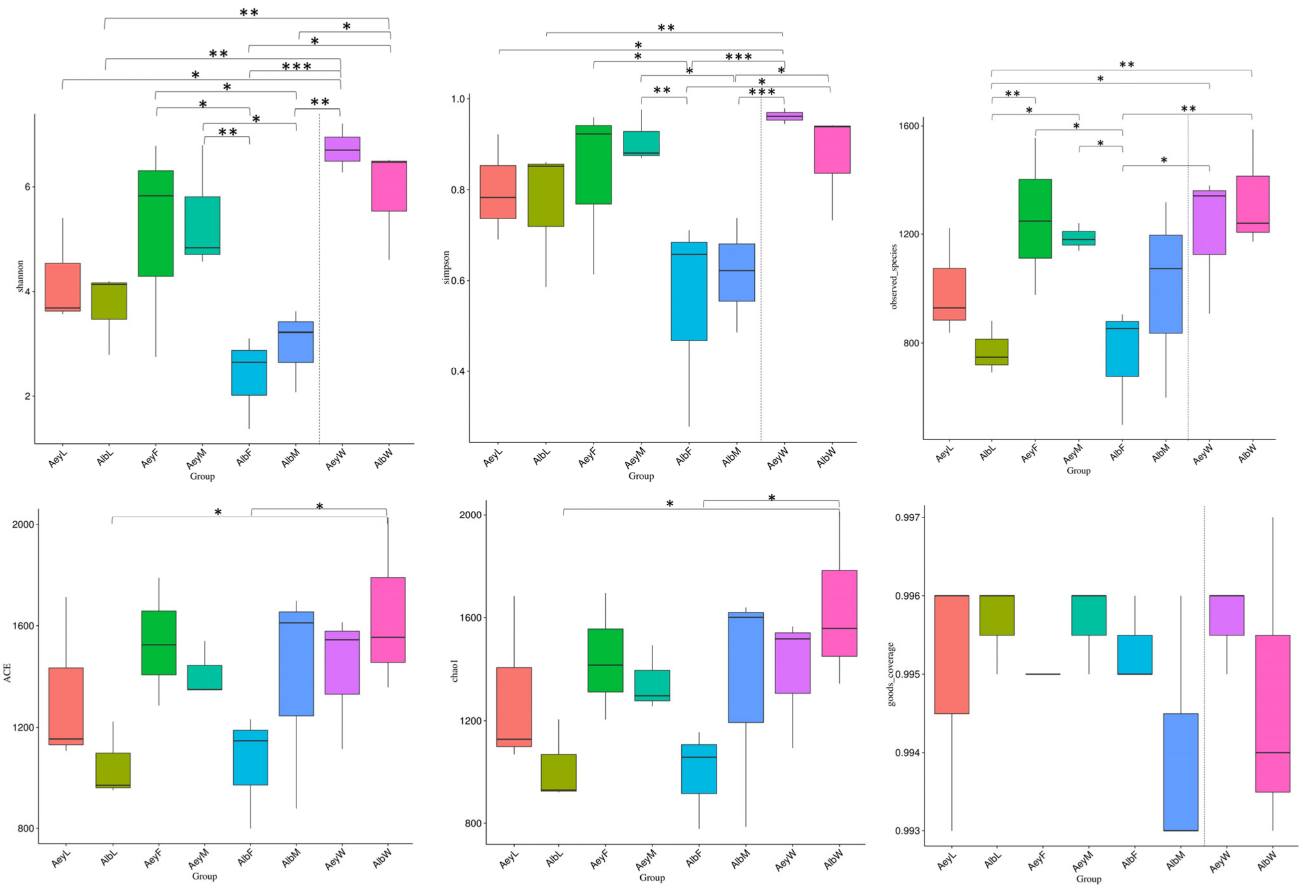

Figure S1: Boxplot of alpha diversity indices. Shannon and Simpson indices reflect the OTU diver-sity in samples. The observed species, ACE and Chao1 indices estimate the OTU richness in samples. Good's coverage estimator represents the percent of the total species represented in each sample category. Wilcoxon and Tukey' s tests were used to detect statistically significant differences between categories (*, p ⩽ 0.05; **, p ⩽ 0.01; ***, p ⩽ 0.001).
